# Influence of Pretreatments on the Conductivity of Flexographic Printed Electronics on Flexible Substrates

**DOI:** 10.3390/polym17233191

**Published:** 2025-11-29

**Authors:** Rocío Silvestre, Raúl Llinares Llopis, Cristian Ariel Olguín Pinatti, Josué Ferri, Ignacio Montava, Eva Bou-Belda

**Affiliations:** 1 Asociación de Investigación de la Industria Textil y Cosmética (AITEX), 03801 Alcoy, Spain; rsilvestre@aitex.es; 2 Departamento de Comunicaciones, Universitat Politècnica de València, 03801 Alcoy, Spain; 3 Instituto Interuniversitario de Investigación de Reconocimiento Molecular y Desarrollo Tecnológico (IDM), Universitat Politècnica de València, 46022 Valencia, Spain; criolpi@upv.es; 4 Centro Integrado Público de Formación Profesional BATOI, 03802 Alcoi, Spain; j.ferripascual@edu.gva.es; 5 Department of Textile and Paper Engineering, Universitat Politècnica de València, Plaza Ferrándiz y Carbonell s/n, 03801 Alcoy, Spain; imontava@upv.es (I.M.); evbobel@upvnet.upv.es (E.B.-B.)

**Keywords:** flexography, e-textiles, wearables, printed-electronics, textiles, electronic textiles

## Abstract

The development of electronic textiles (e-textiles) has advanced significantly thanks to the integration of printing technologies such as flexography, which enables the efficient and reproducible production of conductive circuits on fabrics. This study evaluates the impact of different surface pretreatments (hydrophobic and oleophobic) on the electrical conductivity of flexographically printed circuits on a variety of polyester textile substrates. Key parameters such as grammage, fabric type and surface uniformity are analyzed using stereomicroscopy and profilometry techniques to characterize conductive ink distribution. The results demonstrate that oleophobic pretreatment is more effective at reducing the resulting electrical resistance, promoting better ink adhesion and distribution. Among the fabrics with the best results, those with a more regular and compact structure, such as 15 thread/cm and 666.7 dtex polyester taffeta, show homogeneous ink coverage and the lowest electrical resistance (∼0.5 Ω/cm) compared to more irregular fabrics with discontinuities and higher resistance. The results show that uniformity in ink distribution, assessed by profilometry and color analysis, directly correlates with low electrical resistance. It can be concluded that the combination of a regular and compact textile structure, an adequate surface pretreatment, and a printing direction of the circuit pattern aligned with the weft permits optimizing the conductivity and quality of e-textiles produced by flexography.

## 1. Introduction

Electronic textiles (e-textiles) have become a cornerstone in the development of wearable technologies, enabling garments to integrate sensing, communication, and energy functionalities in a seamless and unobtrusive manner. From early implementations using conductive threads for basic functions such as thermal regulation [1,2], physiological monitoring [3,4], pressure sensing [5] or antenna integration [6,7], the field has evolved toward more sophisticated and integrated systems. These initial approaches, while effective for proof-of-concept and low-complexity applications, presented limitations in terms of circuit resolution, reproducibility, and scalability, especially when considering industrial production and long-term durability.

The need for more versatile, scalable, and precise fabrication methods has led to the adoption of printed electronics as a key enabling technology for e-textiles. Printed electronics allow for the direct deposition of conductive inks onto flexible substrates, offering a lightweight, low-profile alternative to traditional wiring and component integration. Their compatibility with textile substrates has opened new possibilities for integrating sensors [3,8], displays [9,10], and energy systems [11] directly into garments, supporting applications in health monitoring [12,13], sports performance [14], and interactive interfaces [15,16].

A wide range of printing techniques has been explored for this purpose, each with distinct advantages and constraints. Conventional methods such as screen printing and inkjet printing are widely used due to their accessibility and compatibility with various ink formulations. Screen printing is valued for its simplicity and ability to deposit thick ink layers, making it ideal for robust conductive paths [17,18,19]. It has been successfully applied in textile sensors and capacitive interfaces [20]. The process involves forcing ink through a mesh stencil onto the substrate, which allows for high ink volume transfer but limits resolution. In textile applications, screen printing benefits from its ability to accommodate the rough and porous nature of fabrics, although it requires careful control of ink viscosity and curing conditions. Inkjet printing, on the other hand, offers high precision and material efficiency [21,22], enabling fine patterning with minimal ink waste. However, it demands smooth surfaces and is sensitive to ink viscosity and surface energy, often necessitating substrate pretreatments or modifications [23,24].

Other printing techniques, such as aerosol jet printing, 3D printing, and transfer printing, offer additional capabilities but are generally less suited for large-scale textile production. Aerosol jet printing enables deposition on non-planar surfaces with fine resolution [25], though it is costly and technically demanding. Three-dimensional printing allows for multilayered and customized structures [26,27], and its ability to print directly onto curved or textured fabrics makes it attractive for specialized applications. Nevertheless, its slower speed, limited ink compatibility, and higher equipment costs restrict its use in industrial contexts. Transfer-based methods, including thermal transfer printing, are useful for integrating pre-fabricated circuits onto textiles [28]. These techniques facilitate the inclusion of microchips and passive components but often involve multiple steps and may compromise durability under mechanical stress or washing.

Flexographic printing is a particularly promising technique for the industrial-scale production of printed electronics on textiles. Originally developed for packaging, flexography offers high-speed processing, fine pattern resolution, and controlled ink deposition [29,30,31]. Its compatibility with roll-to-roll manufacturing and flexible substrates makes it particularly attractive for e-textile applications. Unlike other methods, flexography balances throughput and precision, making it suitable for large-scale production where consistency and cost-effectiveness are essential. Flexographic printing operates by transferring ink from an anilox roller to a flexible photopolymer plate, which then prints the pattern onto the substrate. The anilox roller plays a central role in controlling the ink volume: its engraved cells determine how much ink is transferred, and its geometry affects the uniformity and thickness of the printed layer. In textile applications, where surface roughness and porosity vary significantly, selecting the appropriate anilox configuration is crucial to achieving continuous and conductive traces.

Compared to other printing techniques, flexography offers a unique balance between resolution, throughput, and compatibility with textile substrates. Screen printing achieves very low sheet resistance (<0.5 Ω/sq) and excellent durability (up to 50 washing cycles) but requires thick ink layers [32,33] and is constrained by its resolution (typically 100–500 µm), making scalability moderate [34]. Inkjet printing provides fine patterns (50–100 µm) but usually yields higher resistance (10–50 Ω/sq), reduced durability (5–10 washing cycles), and limited scalability due to slow processing and substrate preparation requirements [35,36,37,38]. Aerosol jet printing enables features below 50 µm with resistances in the 1–10 Ω/sq range, but its high cost and low throughput restrict widespread industrial adoption [25,39]. Three-dimensional (3D) printing allows multilayer structures and good mechanical durability but typically exhibits high linear resistances above 10 Ω/cm and suffers from very low scalability because of slow deposition rates [40,41]. In contrast, flexography combines moderate resolution (≈100–150 µm) with low linear resistance (≈0.5–2 Ω/cm under optimized conditions), high reproducibility, and compatibility with roll-to-roll manufacturing, achieving durability above 30 washing cycles when combined with appropriate pretreatments [15,42].

Despite its advantages, applying flexography to textiles presents challenges due to the fibrous and heterogeneous nature of fabrics, which can disrupt ink transfer and compromise electrical continuity [11,15]. Achieving reproducible and low-resistance conductive paths on textiles requires careful optimization of both the substrate and the printing process. The physical structure of the textile, such as weave type, yarn density, and grammage, plays a critical role in ink distribution and, therefore, in electrical performance. Fabrics with compact and regular structures tend to support more uniform ink coverage and lower resistance values [43,44,45].

A key strategy for improving print quality on textiles involves modifying the substrate’s surface energy through chemical pretreatments. Hydrophobic and oleophobic coatings have proven effective in enhancing ink adhesion, minimizing spreading, and improving pattern definition by altering the wetting behavior of the fabric and facilitating the formation of continuous conductive traces [46,47]. Building on this foundation, the present study investigates the combined influence of surface pretreatments and textile morphology on the electrical performance of silver nanoparticle inks applied via flexographic printing. A range of polyester fabrics with distinct structural characteristics was analyzed to assess how pretreatment type and fabric architecture affect ink adhesion, distribution, and conductivity. A multiscale characterization approach, including electrical resistance measurements, stereomicroscopy, profilometry, and colorimetric analysis, was employed to correlate surface properties with functional performance and support the development of scalable, reliable, and high-performance e-textiles.

Building on insights from [43] regarding textile structure and composition in conductivity, this study complements those findings by introducing chemical surface modification. Specifically, it systematically evaluates the effect of hydrophobic and oleophobic pretreatments on polyester substrates and their interaction with textile architecture, an aspect not previously addressed. Furthermore, advanced characterization techniques such as optical profilometry and colorimetric analysis are incorporated to establish relations between surface morphology, ink coverage, and electrical performance, extending the methodological scope beyond conventional resistance measurements.

By focusing on industrially relevant polyester substrates and commercially available pretreatment agents, this work aims to identify the optimal combination of textile structure, surface pretreatment, and printing orientation to achieve low electrical resistance and high reproducibility. This integrated approach enhances ink adhesion, reduces variability, and supports industrial scalability.

## 2. Materials and Methods

### 2.1. Materials

Textile Substrates. Based on the results obtained in previous work by Rodes et al. [43], a selection of representative polyester fabrics was made for this study. The chosen materials vary in weave type, thread diameter, and fabric density. Table 1 shows the composition and ligament of the involved fabrics.

The fabric samples were produced by variation in the weft yarn count (333.3 dtex/666.7 dtex) without changing the warp count (167 dtex). The warp density is fixed as well at 60 threads/cm. The value of the linear yarn count is the same for all the fabrics, being 1.5%. Table 2 shows the size and weight of the different fabrics used.

**Figure 1:**
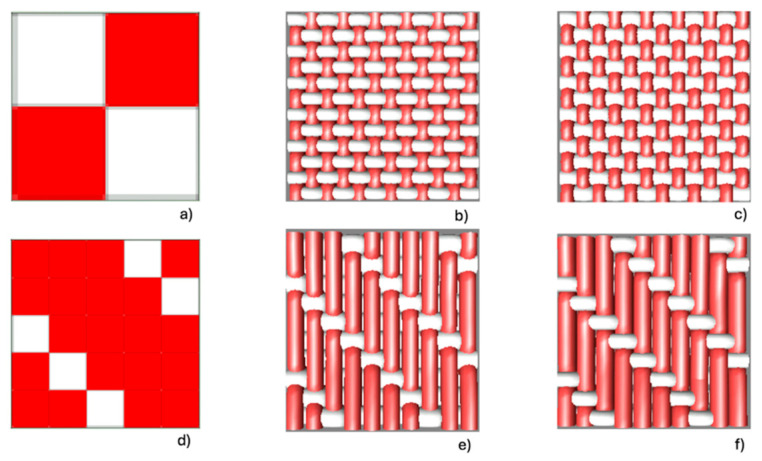
Graphic representation of ligament and fabric simulations of the two structures used: (**a**–**c**) Plain weave. (**a**) Diagram of the plain weave. (**b**) Simulation of the plain weave fabric with 333.3 dtex yarn. (**c**) Simulation of the plain weave fabric with 666.7 dtex yarn. (**d**–**f**) Twill weave. (**d**) Diagram of the twill weave. (**e**) Simulation of the twill weave fabric with 333.3 dtex yarn. (**f**) Simulation of the twill weave fabric with 666.7 dtex yarn.

Figure 1 shows the graphic representation of the ligament and fabric used for the two groups of fabrics, taffeta and twill, varying the weft yarn count.

Pretreatment. Two different pretreatments have been applied to the textile substrates. The first one applies a hydrophobic agent, Smartrepel Hydro CMD liq from Archroma (Pratteln, Switzerland). This agent provides durable water repellence without the use of fluorocarbons, making it an environmentally friendly option. It ensures that the fabric remains breathable and comfortable while offering excellent resistance to water and stains. According to the technical data sheet, Smartrepel Hydro CMD liq presents a pH (5%) of about 5, a density (20°) of about 1.00 g/cm^3^ and a flash point greater than 100 °C. The second pretreatment consists of the application of an oleophobic agent, Nuva N1811 from ARCHROMA. It is a C6-based fluorochemical microencapsulated coating that repels water, oil, alcohol, soil, and stains. After the treatment, the fabric is permeable to air, stays soft with no negative impact on abrasion resistance and tear strength. According to the technical data sheet, Nuva N1811 presents a pH (5%) of about 4–6, a density (20°) of about 1.05 g/cm^3^ and a flash point greater than 100 °C.

Ink. The conductive ink employed in this study was PFI-RSA6004 from Novacentrix (Austin, TX, USA), selected for its high electrical performance (Table 3). This silver nanoparticle-based ink, formulated in an aqueous medium, is specifically designed for printing reflective and conductive patterns on smooth substrates. Its composition and rheological properties make it suitable for flexographic printing processes, including laboratory-scale and roll-to-roll applications.

**Figure 2:**
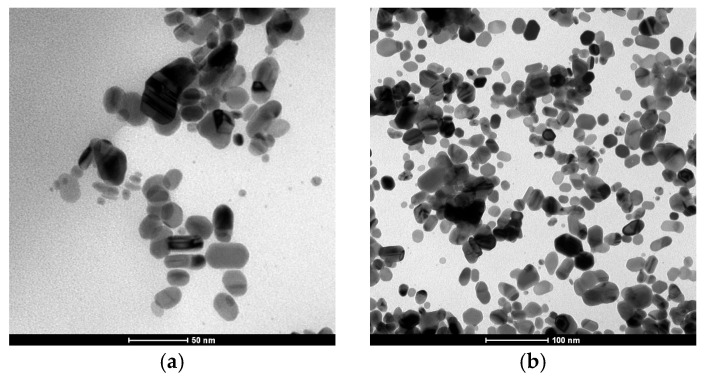
TEM micrograph images of PFI-RSA6004 Silver ink from Novacentrix: (**a**) Image taken at 1,000,000× magnification (scale bar = 50 nm). (**b**) Image taken at 500,000× magnification (scale bar = 100 nm). Images courtesy of Novacentrix.

Transmission Electron Microscopy (TEM) analysis of the ink shows the presence of silver nanoparticles with predominantly irregular to ellipsoidal morphologies. As shown in Figure 2, the particles exhibit sizes mainly in the range of 20–50 nm, with some smaller particles below 20 nm and occasional elongated structures exceeding 50 nm. The high-contrast regions observed within the particles suggest crystalline domains, consistent with metallic silver. These results support the claim of silver nanoparticle incorporation and provide evidence of their nanoscale dimensions and morphology.

### 2.2. Methods

Pretreatment. Hydrophobic and oleophobic pretreatments were applied to the selected fabrics with the purpose of reducing surface tension and modifying surface energy. These modifications were expected to influence the behavior of conductive ink deposition during flexographic printing by limiting ink spreading and promoting more uniform layer thickness. Additionally, such treatments may contribute to improved reproducibility of printed circuits and more consistent electrical performance across different batches and substrates.

**Figure 3:**
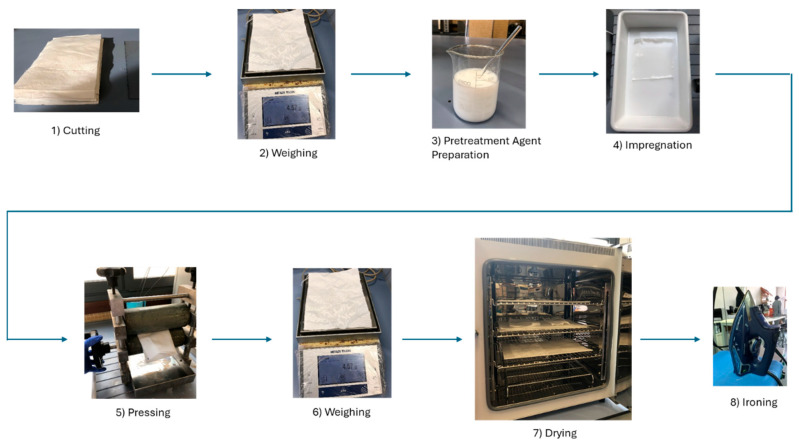
Steps for preparing the substrate textile samples before flexographic printing.

For each one of the substrates, the pretreatment procedure consisted of 8 steps as reflected in Figure 3. Firstly, three samples were cut with a size of 190 × 120 mm approximately (one with no pretreatment for comparison purposes, one for the hydrophobic pretreatment, and the third one for the oleophobic pretreatment). Secondly, the samples were weighed before applying the pretreatment. Next, 400 mL of both pretreatments were prepared according to the manufacturer’s specifications, 60 g/L for the hydrophobic one and 50 g/L for the oleophobic one. The pH of the oleophobic covering was corrected using acetic acid 96% with a concentration of 1 mL/L. Once the pretreatments were ready, the samples were impregnated by submersion in a bucket with each pretreatment for 30 s on each side (right and back). Afterwards, the samples were folded in half and proceeded to pass them through a manual pad to remove the excess of the product. This step was repeated 2 times. Next, the samples were weighed again to estimate the pick-up of the pretreatment. The pick-up indicates the quantity of absorbed chemical products after pretreatment. It is expressed as a percentage (I %) and defined by the following equation:

(1) $$I\left(\%\right)=\frac{Wet\;Weigh-Dry\;Weigh}{Dry\;Weigh}\times 100$$

Finally, the samples were dried and cured using a forced air oven, specifically the ArgoLab TCF 120 oven from Giorgio Bormac (Carpi, Italy). According to the manufacturer, the drying process needs 10 min at 120 °C, and the curing process must be carried out at 150 °C for 3 min. Lastly, before applying flexographic printing, the samples were ironed with an iron in nylon mode (1 black point) to ensure the best printing results.

**Figure 4:**
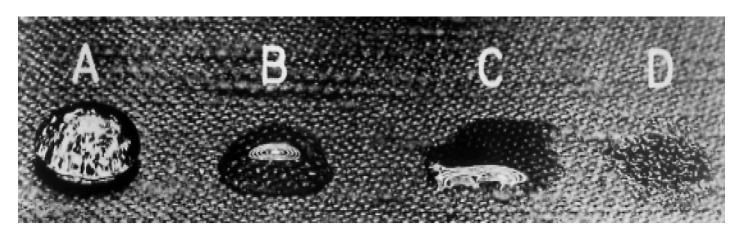
Value scale of drop situation for UNE-EN ISO 14419:2010. A: well-rounded drop. B: rounding drop with partial darkening. C: wicking apparent and/or complete wetting. D: complete wetting.

Once the three samples were prepared, the effectiveness of the applied pretreatment was evaluated using a drop situation test, such as the one used in UNE-EN ISO 14419:2010. The results of the pretreatments were compared with the untreated samples. Figure 4 shows the scale of the evaluation used in UNE-EN ISO 14419:2010.

**Figure 5:**
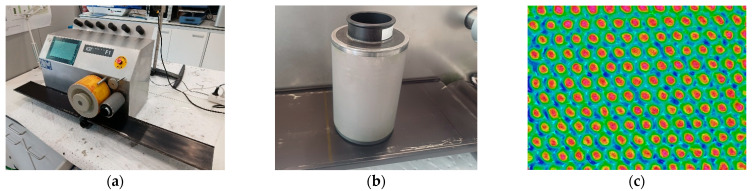
(**a**) Flexographic Printer used in the research. (**b**) Anilox used in the research. (**c**) Anilox cell detail.

Printing. One-layer flexographic prints were carried out on the samples using the IGT F1 from IGT Testing Systems (Almere, The Netherlands). The printing plate used was based on a photopolymer specially developed for printed electronics applications from KodaK (Rochester, NY, USA). Figure 5 shows the flexographic printer, the anilox used in this work and the details of the anilox cells.

**Figure 6:**
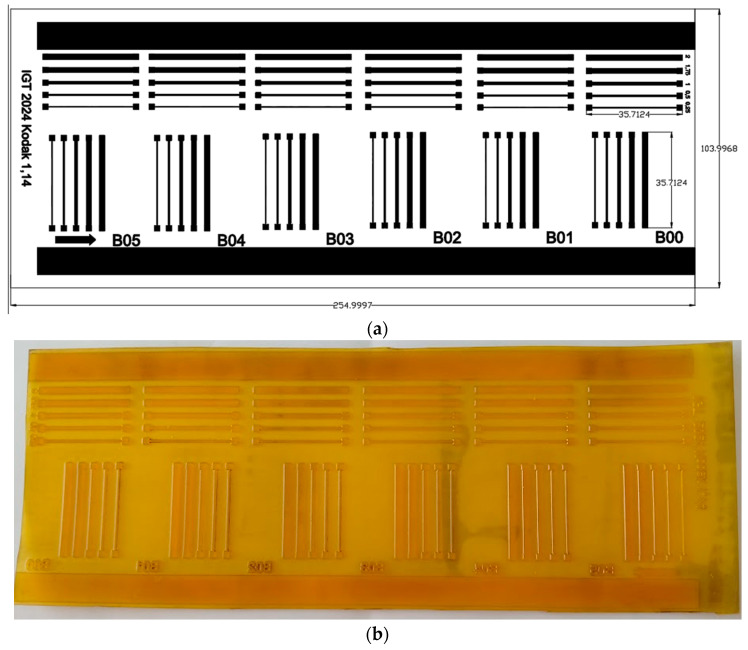
Printing plate applied on the flexographic prints: (**a**) dimensions, (**b**) final aspect of the printing plate.

The print patterns were designed as linear elements oriented at 0° and 90°, with varying line widths to evaluate their influence on electrical resistance (Figure 6). The minimum line width was established based on the thickest standard used by printed circuit board (PCB) manufacturers, Class 3, which corresponds to 0.25 mm. Then, the width is increased to 2 mm. This selection aims to assess the feasibility of achieving standardized printed circuits on textile substrates.

Furthermore, the printing plate incorporates the full range of microstructures supported by KodakK, from B00 to B05. Among these, the B05 microstructure enables the highest ink transfer to the textile substrate. Ink transfer is a critical factor in achieving consistent conductivity and print quality, and, therefore, plays a key role in the overall performance of printed e-textiles.

The process began with the cleaning of the equipment prior to use to ensure the removal of any residual inks, dust, or contaminants that could interfere with print quality. The printing plate was cleaned with acetone to prevent clogging, while the anilox area was treated using enpurex Cleaning Liquid Power from TKM (Remscheid, Germany). Following this, the flexographic printing machine was configured according to the parameters listed in Table 4. To optimize the outcome, several secondary parameters were adjusted during printing trials, yielding the best results under the following conditions:

- Anilox Force: 150 N.
- Anilox Speed: 50%.
- Printing Force: 500 N.

Electrical characterization. Resistance measurements were made using a four-point probe configuration (Kelvin method) to minimize contact resistance effects, ensuring high accuracy and repeatability. A Keithley 2001 Series Digital Multimeter from Tektronix (Beaverton, OR, USA), a high-precision and low-noise-floor device, was employed to perform the resistance measurements. The probes were aligned to guarantee consistent spacing and contact pressure across all samples. For each sample, five consecutive resistance measurements were recorded to evaluate both repeatability and statistical dispersion.

Stereomicroscopy. Microstructural analysis of the fabric samples and printed ink was carried out using a MZ APO stereomicroscope from Leica (Wetzlar, Germany), equipped with a fully apochromatically corrected optical system. Samples were positioned on a flat, stable support to ensure consistent focus and minimize optical distortion. A zoom range from 8× to 80× was employed to examine the spatial distribution of the threads, the weave structure, and the interaction between the ink and the textile surface. The system’s high numerical aperture (up to 0.2) enabled detailed visualization of fine features, supporting the qualitative assessment of thread alignment, fabric uniformity, and ink deposition.

Profilometry. Surface topography and geometric characterization of the fabric samples were conducted using a Profilm3D desktop optical profiler from KLA (Milpitas, CA, USA), which operates based on white light interferometry (WLI). Prior to measurement, the samples were mounted on the stage using a flat, non-reflective support to ensure stability and minimize light scattering. An objective 50× was used, enabling the determination of the geometric characteristics of the fabric, the sizes of the threads, their separation and arrangement of the weft and warp. Scans were performed over regions of interest, capturing representative areas of the fabric structure. Multiple scans (*n* = 5) were conducted at different locations on each sample to evaluate structural uniformity.

Color Measurement. Color measurements of the treated samples were performed using a CM-3600d reflectance spectrophotometer from Konica Minolta (Tokyo, Japan). The instrumental geometry was d/8 with the specular component excluded. The measurement area had a diameter of 25.4 mm. Ultraviolet energy was included. Measurements were carried out using the CIE 10° standard observer and the D65 standard illuminant over the wavelength range of 400–700 nm.

## 3. Results and Discussion

### 3.1. Pretreatments

Pick-Up. Pick-up values obtained after pretreatment are shown in Table 5. Both excessive and insufficient penetration can compromise the subsequent ink adhesion: deeper penetration may reduce the amount of treatment available at the surface for interaction with the ink, while shallow penetration may result in incomplete coverage of the fibers. Therefore, precise control of pick-up is essential to ensure that the surface is adequately modified for optimal ink deposition in the following printing step.

**Figure 7:**
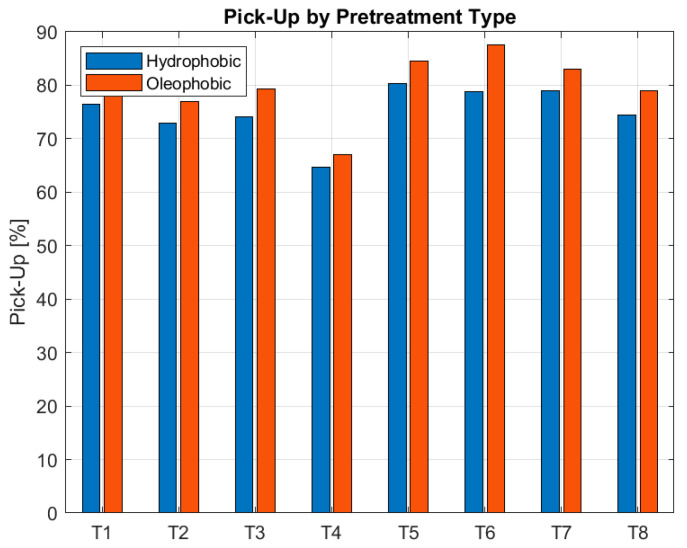
Comparison of Pick-Up percentage across textile substrates T1–T8 under hydrophobic and oleophobic surface treatments.

Grouped bars in Figure 7 represent the percentage of treatment uptake for each fabric sample, highlighting differences in liquid absorption depending on substrate and type of pretreatment.

The pick-up results for hydrophobic and oleophobic pretreatments reveal a clear influence of both fabric structure and weave type on treatment absorption. Among all polyester fabrics studied, those with a plain weave and high weft density, T2 (72.87% hydrophobic, 77.02% oleophobic) and T4 (64.73%, 66.98%), consistently exhibit the lowest pick-up values. This behavior can be attributed to the combination of a tightly packed structure and the inherently closed nature of the plain weaves, which significantly restricts pore accessibility and internal capillarity, minimizing pretreatment penetration and retention.

In contrast, twill weave fabrics (T5–T8), characterized by their diagonal structure and increased porosity, show higher pick-up values overall. T5 (80.26%, 84.47%) and T7 (79.02%, 82.94%), both with low weft density, display the highest absorption due to their open structure and larger pore channels. T6 (78.80%, 87.51%), despite its high density, shows the highest oleophobic absorption, suggesting that yarn fineness and internal capillarity may override the density effect. T8 (74.39%, 78.92%), with high density and coarse yarn, stands out as the least absorbent twill fabric, though it still absorbs more than the most compact plain weaves.

These findings confirm that weave type can be a decisive factor in controlling pretreatment uptake: no twill fabric reaches the low absorption levels of the most compact plain weaves. Moreover, low pick-up is achieved only when both high weft density and a closed weave structure coincide, as in T2 and T4.

Drop Situation Test. The results of the drop test are presented in Table 6, following the assessment criteria of UNE-EN ISO 14419:2010, where repellence is graded from A (highest repellence) to D (lowest repellence).

Most of the pretreated fabrics, regardless of whether hydrophobic or oleophobic agents were used, achieved the highest repellency rating (A), confirming the effectiveness of the applied pretreatments in modifying surface tension and enhancing liquid resistance. Notably, sample T8 treated with oleophobic agents exhibited a reduced performance (B rating), likely due to structural irregularities of the textile, which prevented uniform distribution of the agent.

**Figure 8:**
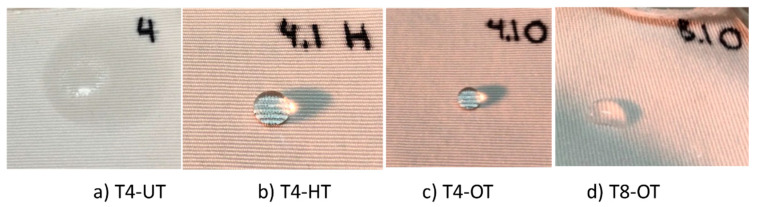
Drop Situation results for (**a**) T4-UT (Untreated), (**b**) T4-HT (Hydrophobic Pretreatment) and (**c**) T4-OT (Oleophobic Pretreatment). Figure (**d**) shows the worst result for a pretreatment (T8-OT).

Figure 8 shows the results for T4 for the cases studied, no pretreatment and both pretreatments. The last image of Figure 8, (d), shows the results for T8 with oleophobic pretreatment, where the drop does not have as much consistency as in the other cases shown.

All treatment formulations demonstrated an adequate level of water repellence across the available substrates. These results suggest that further optimization of the formulation could be explored to reduce production costs and improve process efficiency, without compromising functional performance.

### 3.2. Flexographic Printings

Printings. Before printing, each fabric was cut into two distinct orientations. One sample had its longer axis aligned with the weft direction (vertical yarns), while the other was aligned with the warp direction (horizontal yarns). Each one of two samples of each fabric was printed once using flexographic printing. For the first sample, printing was performed with the printing direction aligned to the weft. For the second sample, the printing direction was aligned with the warp, following the horizontal yarn orientation. This setup enables evaluation of how fabric orientation relative to the printing direction influences ink deposition, image definition, and functional properties such as electrical resistance and color uniformity.

**Figure 9:**
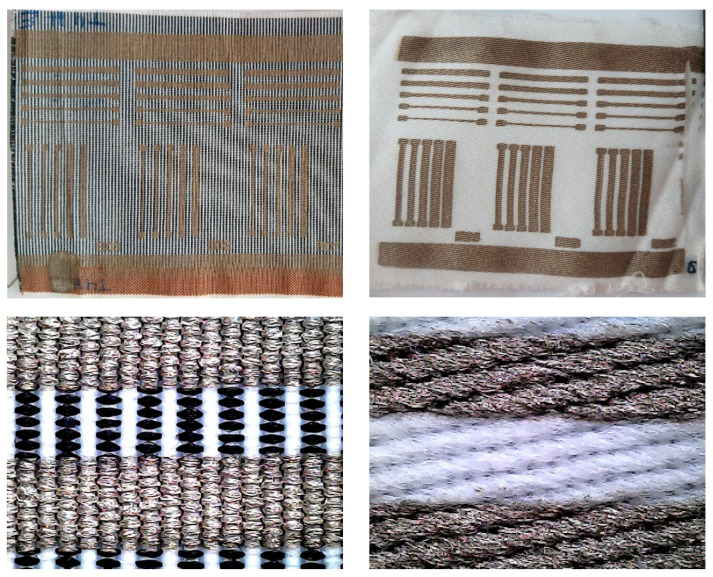
Example of pattern printing on a textile substrate. (**Left**) T4_HT printed on weft direction. (**Right**) T8_HT printed on warp direction.

Figure 9 shows two examples of flexographic printing with silver conductive ink on two different fabrics cut in the warp orientation. The differences in printing will be analyzed in terms of the resistance values obtained.

### 3.3. Electrical Characterization

**Figure 10:**
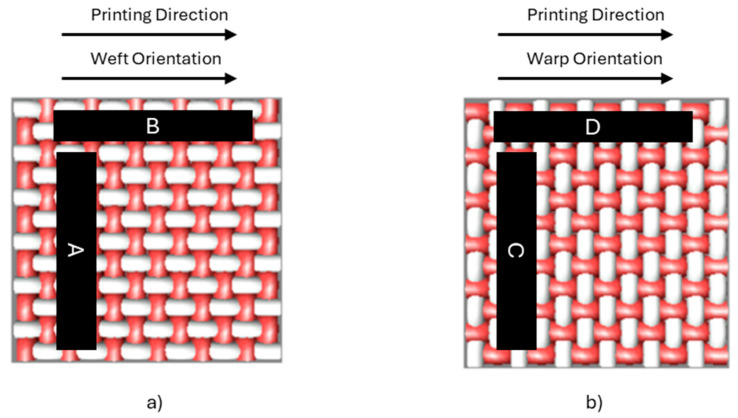
Designation of sample labels according to printing direction: (**a**) alignment with warp orientation; (**b**) alignment with weft orientation.

Resistance measurement. Measurements were performed considering the printing direction relative to the weft and warp orientations. Figure 10 illustrates the nomenclature applied according to the printing alignment. When the printing direction corresponds to the weft orientation, sample A refers to the measurement taken on a print oriented toward the warp, while sample B corresponds to the print aligned with the weft. Conversely, when the printing direction is aligned with the warp orientation, sample C represents the measurement on the print oriented toward the weft, and sample D corresponds to the one aligned with the warp.

For each printing, the minimum line width at which electrical conductivity was achieved was first identified. Fabrics T1 and T3 only exhibited conductive prints for sample B in the case of the Hydrophobic Pretreatment (in both fabrics) and for the same sample, B, for the Oleophobic Pretreatment (only in fabric T3). In all three cases, conductivity was obtained exclusively on the line with the largest width, 2 mm. The remaining fabrics achieved conductivity at widths of 1.75 mm and 2 mm. For the 1.75 mm width, the lower one, five resistance measurements were taken for the line in the horizontal direction (sample B or D in Figure 10, depending on whether the printing direction aligns with the weft or warp orientation) and for the vertical line (sample A or C). These ten measurements were repeated for the six microstructures (B00 to B05). The procedure was applied to samples printed in both weft and warp directions, which yielded 30 measurements for each combination of printing direction and fabric orientation (A, B, C, and D). The mean and standard deviation were then calculated from these measurements.

Table 7 summarizes the average electrical resistance values (Ω) along with the standard deviation obtained from each textile sample as a function of printing direction and fabric orientation (weft vs. warp), under three surface conditions: untreated (UT), oleophobic pretreatment (OT), and hydrophobic pretreatment (HT). The nomenclature follows the schema defined in Figure 10, where printing direction and fabric orientation combinations are labeled A–D for each pretreatment. Values marked as ‘–’ indicate high impedance or open circuit conditions. This approach provides a comprehensive overview of the electrical behavior of each textile sample under different printing and surface treatment conditions. These averages provide a reliable basis for comparing fabrics and treatments, particularly in this exploratory context aimed at identifying general trends in conductivity improvement.

T1 and T3 exhibit open circuit behavior when untreated or after oleophobic treatment, only showing occasional tens to hundreds of ohms under hydrophobic treatment. T2 and T4 exhibit very low resistances (<10 Ω) after oleophobic pretreatment in all orientations, indicating excellent ink adhesion and conductivity. The surface tension of the fabrics achieved using the oleophobic agent facilitates better adhesion and distribution of the conductive ink, resulting in lower electrical resistance. T5 and T6, which initially showed resistance in the hundreds of ohms when untreated or oleophobic-treated, drop dramatically to around ten ohms with hydrophobic treatment. T7 displays an anomalous spike above 10^4^ Ω under hydrophobic treatment, suggesting over-treatment or poor ink–substrate compatibility. Finally, T8 transitions from very high resistance (>10^5^ Ω) when untreated to single-digit ohms after oleophobic treatment, then rises again under hydrophobic treatment, underscoring its strong sensitivity to pretreatment type.

Although pretreatment absorption (pick-up) could be expected to influence conductivity, regression analysis between pick-up values (Table 5) and electrical resistance (Table 7) for oleophobic pretreatment yielded a weak correlation (R^2^ ≈ 0.23). The computed linear model was:

(2) $$Resistance\;\left(\Omega \right)=\;\;6.77\;\times Pick-Up\;(\%)-\;480.66\;$$

While some individual cases appear to suggest a trend, such as T4 (66.98%, ≈0.52 Ω/cm) compared to T5 (84.47%, ≈262 Ω/cm), the overall variability across fabrics indicates that absorption alone does not explain conductivity. For hydrophobic pretreatment, the correlation was even lower (R^2^ ≈ 0.05). Therefore, pretreatment pick-up cannot be considered a primary determinant of electrical performance.

The printing direction relative to the weft and warp of the fabric influences electrical resistance. When the printing direction of the circuit lines coincides with the weft direction, the best results are obtained (lower electrical resistance) for T4. Fabrics with a more regular and compact structure (such as T4) allow for a more homogeneous and continuous distribution of the conductive ink, which reduces electrical resistance. This aspect will be further investigated through microscopy and profilometry analyses to assess surface uniformity and roughness in fabrics T2 and T4.

**Figure 11:**
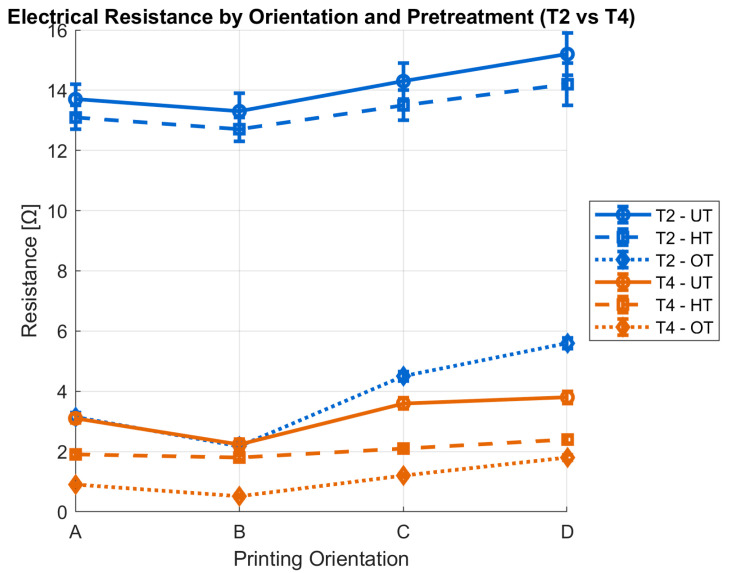
Electrical resistance of substrates T2 and T4 as a function of printing orientation (A–D) and surface treatment. Error bars represent standard deviation from multiple replicates.

Subsequent work will focus exclusively on substrates T2 and T4. These two samples were selected because they alone combine consistently low and reproducible electrical resistances across all printing orientations (A–D) and surface treatments. Figure 11 graphically illustrates the relationship between the measured electrical resistance and the printing orientation, as well as the applied surface treatment, for T2 and T4 textile substrates. The best results are obtained with T4 fabric when the print direction coincides with the weft direction, and the circuit line pattern is aligned with it. Error bars indicate standard deviation from four measurements per condition, showing variability across orientations.

**Figure 12:**
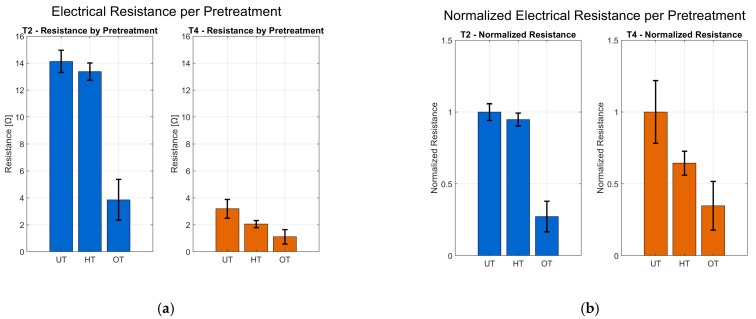
(**a**) Electrical resistance for substrates T2 and T4 under untreated (UT), hydrophobic (HT), and oleophobic (OT) conditions, averaged across four printing orientations (A–D). (**b**) Normalized electrical resistance relative to the untreated condition for each substrate and treatment. Error bars represent standard deviation from four measurements per treatment.

Electrical resistance decreases with oleophobic pretreatment compared to hydrophobic pretreatment and to unpretreated samples. This is clearly observed in Figure 12, where (a) shows the absolute resistance values and (b) presents normalized resistance relative to the untreated condition. In both pretreatments, the resistance of printed linear elements on T4 (oleophobic) is significantly lower than on all samples of T2 and lower than on T4 untreated samples, confirming the effectiveness of the oleophobic treatment. Error bars in both subfigures indicate variability across four orientations (A–D).

### 3.4. Physical Characterization

**Figure 13:**
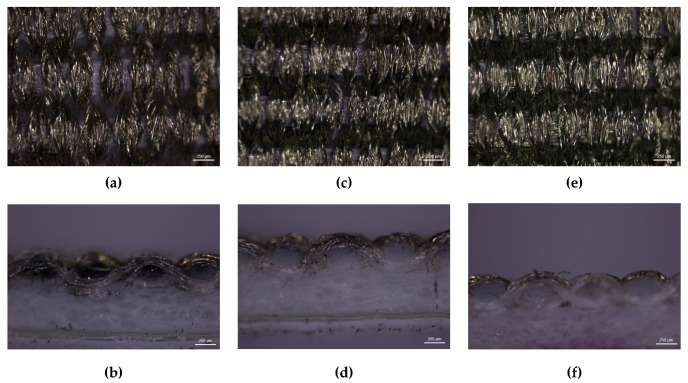
Surface (**top**) and cross-sectional (**bottom**) views of sample B of substrate T2 under different treatments (UT—Untreated, (**a**,**b**); HT—Hydrophobic treatment, (**c**,**d**); OT—Oleophobic treatment, (**e**,**f**)).

**Figure 14:**
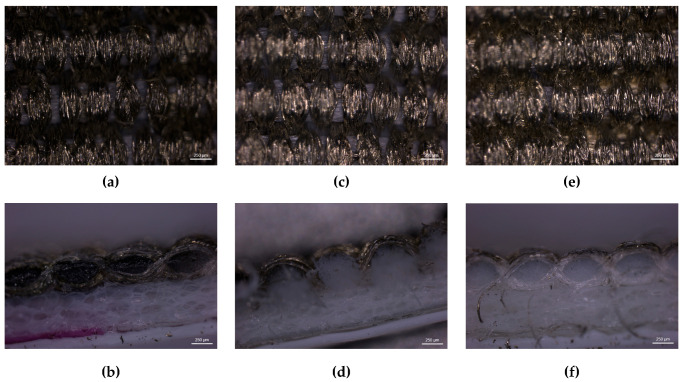
Surface (**top**) and cross-sectional (**bottom**) views of sample B of substrate T4 under different treatments (UT—Untreated, (**a**,**b**); HT—Hydrophobic treatment, (**c**,**d**); OT—Oleophobic treatment, (**e**,**f**)).

Stereoscopic Microscopy. Stereoscopic microscopy evaluated flexographic printing quality on both textile substrates. The analysis focused on samples printed in the weft direction (sample B). Figure 13 and Figure 14 present surface and cross-sectional views of the weft-oriented prints on fabrics T2 and T4, respectively.

The stereomicroscopy and cross-sectional views of T2 reveal an irregular ink distribution, particularly in the untreated (UT) condition. Ink penetration varies depending on the treatment applied, with the oleophobic treatment resulting in the least penetration. This penetration is inconsistent across the treatments, with visible uncoated pores and fragmented conductive paths. Hydrophobic treatment (HT) slightly improves surface coverage with respect to UT, but still results in uneven ink deposition and accumulation zones, indicating poor compatibility between the treatment and the textile structure. Oleophobic treatment (OT), while more effective, still struggles to achieve full uniformity due to the fabric’s inherent irregularity. Interestingly, cross-sectional observations suggest that lower penetration may favor electrical performance, as reflected in the electrical resistance values: UT and HT samples show resistances around 13.3 Ω/cm and 12.7 Ω/cm, respectively, while OT reduces resistance to approximately 2.17 Ω/cm.

T4 demonstrates much more favorable behavior across all treatments. The untreated sample already shows better ink coverage than T2, thanks to its compact and regular structure. Hydrophobic treatment (HT) enhances this further, yielding a more continuous ink layer with fewer discontinuities. Oleophobic treatment (OT) provides the best results, enabling uniform ink penetration and forming uninterrupted conductive traces. The cross-sectional analysis confirms consistent ink distribution throughout the textile thickness. As observed in T2 analysis, the sections suggest that controlled penetration, rather than excessive absorption, correlates with improved conductivity, reinforcing the importance of balanced ink interaction with the substrate. These structural advantages translate into significantly lower electrical resistance values: UT samples measure around 2.24 Ω/cm, HT samples drop to 1.80 Ω/cm, and OT samples achieve the lowest resistance at approximately 0.52 Ω/cm. The superior performance of T4 is directly linked to its dense weave and effective interaction with the oleophobic agent.

The comparison between T2 and T4 highlights the critical role of textile structure and surface treatment in determining ink absorption and electrical performance. While both fabrics benefit from oleophobic treatment, only T4 achieves optimal conductivity due to its compact and uniform morphology. The more irregular weave of T2 limits the effectiveness of both pretreatments. Oleophobic treatment consistently outperforms hydrophobic and untreated conditions in both substrates, but its impact is maximized when paired with a structurally favorable fabric like T4. These findings underscore the importance of combining appropriate surface chemistry with textile engineering to optimize flexographic printing for e-textile applications.

Profilometry. The use of two different weft yarn thicknesses (333.3 dtex for T2 and 666.7 for T4) has a direct influence on pretreatment as well as on the posterior ink deposition, even when the fabric weave structure remains the same (plain for T2 and T4). This variation in weft yarn modifies the surface topography of the textile substrate, which significantly impacts the continuity and uniformity of the conductive ink deposited through flexographic printing.

**Figure 15:**
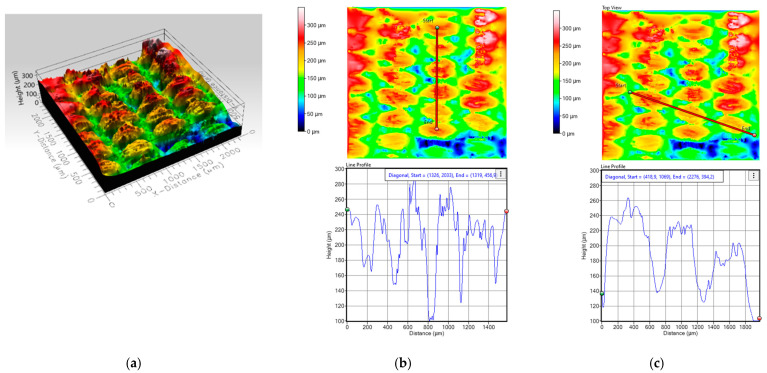
(**a**) Profile of the T2 substrate, (**b**) profile in the warp direction and (**c**) profile in slope in the weft direction.

**Figure 16:**
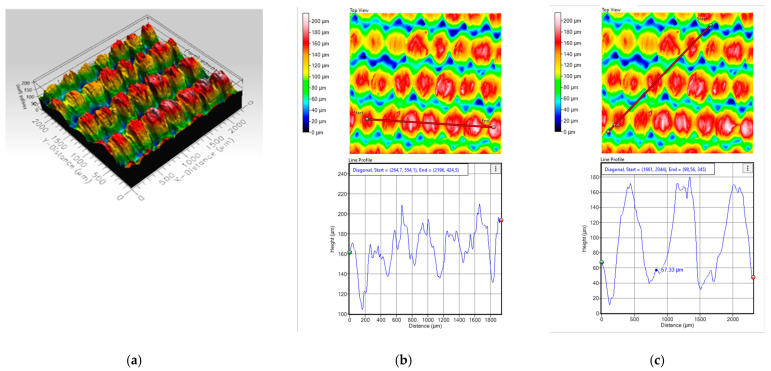
(**a**) Profile of the T4 substrate, (**b**) profile in the warp direction and (**c**) profile in slope in the weft direction.

To investigate this influence, this section analyzes oleophobic pretreated samples of T2 and T4 printed in the weft direction. Using optical profilometry techniques, a comparison of the surface morphology in both the weft and warp directions is carried out. Figure 15 and Figure 16 show the topographic images obtained for each printed fabric, allowing the evaluation of key parameters such as regularity, thread spacing, and surface roughness, all of which are directly related to the quality and functionality of the resulting conductive traces.

The profilometric analysis of substrate T2 reveals a highly irregular surface morphology in both warp and weft directions. The 3D topography image shows alternating zones of red (≈250 µm), yellow (≈200 µm), and blue (≈50 µm), confirming the presence of abrupt height changes and a lack of surface uniformity. These color transitions visually reinforce the interpretation of high surface roughness and discontinuity, which negatively affect the formation of conductive traces. In the warp direction, peak heights reach 280 µm and 240 µm, with peak-to-valley differences ranging from 180 µm to 80 µm (100 µm of variation), depending on the region. This irregularity correlates with the electrical resistance measured in this direction under oleophobic treatment (A-OT ≈ 3.2 Ω/cm), indicating poorer ink continuity. In the weft direction, the surface remains uneven, with heights of 260 µm, 220 µm, and 200 µm, and peak-to-valley differences of 120 µm and 80 µm (40 µm of variation). The corresponding electrical resistance (B-OT ≈ 2.2 Ω/cm) reflects the impact of these topographical variations, which hinder uniform ink deposition and reduce conductivity.

Substrate T4 presents a significantly more regular and compact surface based on profilometric results. The 3D topography image is predominantly red, indicating a smooth and consistent surface with minimal height variation. This visual pattern confirms a uniform and compact surface, which facilitates better ink distribution and enhances conductivity. In the warp direction, excluding the initial peak, the surface heights stabilize around 200 µm and 180 µm, with consistent peak-to-valley differences of approximately 40 µm. This uniformity supports the formation of continuous conductive paths, resulting in a low electrical resistance (A-OT ≈ 0.9 Ω/cm) in this orientation. In the weft direction, the surface is even more homogeneous, with heights around 160 µm and peak-to-valley differences of ~100 µm, contributing to the lowest resistance observed (B-OT ≈ 0.5 Ω/cm).

The comparison between fabric printed results in T2 and T4 highlights the critical influence of surface topography and regularity on electrical performance. Substrate T2 exhibits pronounced irregularities and deep inter-yarn valleys, which disrupt ink continuity and lead to higher resistance values in both warp and weft directions. In contrast, substrate T4 demonstrates a smoother and more uniform surface, with shallower and consistently spaced valleys that facilitate better ink penetration and trace formation. Additionally, the weft direction consistently shows greater regularity than the warp direction, which is reflected in the lower resistance values obtained in weft-aligned prints for both substrates. The profilometric and colorimetric data directly support the electrical measurements, showing that lower surface roughness and higher regularity, especially in the weft direction, are essential for achieving low resistance and reliable conductivity in flexographically printed e-textiles.

Color Measurements. To evaluate the color obtained on the surface of the fabric treated by flexography, depending on the type of fabric and the pretreatments applied, the color of the samples was measured to investigate whether there is a relationship between the hue and lightness or darkness of the color and the resulting resistivity. Table 8 shows the colorimetric coordinates (L*, a*, b*) in the CIELAB color space, as well as the color difference with respect to the reference sample.

The relationship between the resistance value of the samples and the color measurements is obtained with an R2 of 0.61, an adjusted R2 of 0.22, and a mean absolute error of 2.97, with the variables used being L*, a* and b*. Equation (3) shows this correlation.

(3) $$R=120.02-3.05\cdot L^{*}+7.05\cdot a^{*}+4.49\cdot b^{*}$$

**Figure 17:**
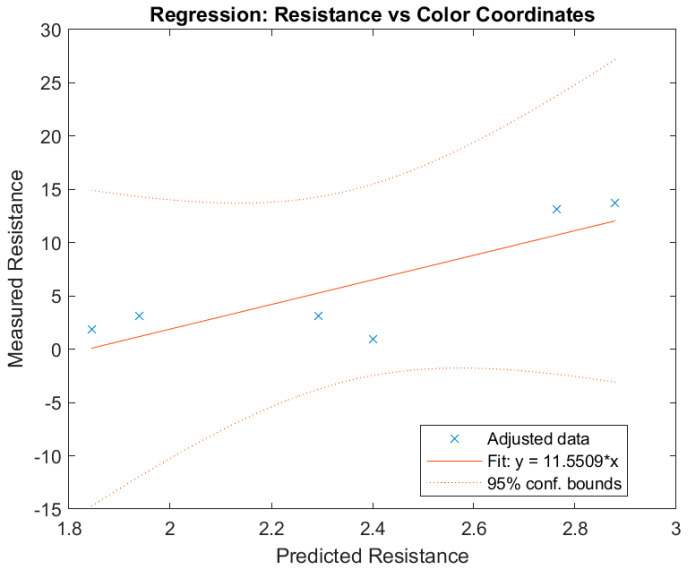
Regression analysis correlating CIELAB color coordinates (L*, a*, b*) with measured electrical resistance of textile samples.

The scatter plot in Figure 17 compares predicted resistance values from the regression model against experimentally measured data. The diagonal dashed line represents the ideal fit (predicted = measured). Deviations from the line indicate estimation error and reflect sample-specific variability related to ink coverage and substrate structure. This model demonstrates a moderate correlation (R^2^ = 0.61), suggesting that colorimetric measurements may serve as a complementary predictor of conductivity in printed fabrics. While the relationship is not perfect, a consistent trend is observed: increased ink deposition, reflected in greater color change, tends to result in lower electrical resistance. This moderate correlation is expected because colorimetry describes only the optical surface properties of the printed trace. Electrical performance, however, depends on additional microstructural factors such as porosity, surface roughness, yarn density, and, importantly, the depth-wise penetration of the conductive ink. These parameters influence the continuity and effective cross-section of the conductive path and are not captured by colorimetric data, thereby limiting the achievable correlation.

On substrates such as T4, where ink coverage is homogeneous (as shown by microscopy and profilometry images), color measurement is also more uniform, resulting in less variability in resistance between samples. In contrast, on fabrics such as T2, where there are areas with less ink (less intense color), higher resistance and greater dispersion in the electrical results are observed. The correlation suggests that other factors, such as ink penetration into the textile structure or the presence of voids between threads, also influence conductivity and are not always reflected in the surface color measurement.

Objective color measurement complements physical and electrical characterization, since while microscopy and profilometry show the surface distribution and morphology of the ink, colorimetry provides quantitative data that is easy to compare and reproduce, useful for both laboratory quality control and industrial processes.

## 4. Conclusions

This study demonstrates that the combination of textile structure and surface pretreatment is critical for optimizing the electrical performance of flexographically printed e-textiles. The textile structure governs pretreatment effectiveness and determines ink deposition uniformity. Optimal electrical performance requires careful selection and matching of both pretreatment and textile architecture.

Oleophobic pretreatment consistently outperforms hydrophobic and untreated conditions, enabling lower electrical resistance by promoting better ink adhesion and more uniform distribution, especially when applied to fabrics with compact and regular morphology.

Profilometric analysis reveals that surface regularity and reduced roughness are essential for achieving continuous conductive traces. Substrates with higher weft yarn thickness (such as T4) exhibit shallower and more uniform valleys, which facilitate effective pretreatment absorption and homogeneous ink deposition. In contrast, fabrics with irregular and rough surfaces (such as T2) show deeper valleys and greater topographical variability, resulting in higher resistance values due to discontinuities in the printed paths.

A direct comparison between warp and weft directions further highlights the importance of structural regularity: the weft direction, which is more regular and compact, consistently yields lower electrical resistance than the warp direction for both substrates. These findings confirm that achieving low resistance and reliable conductivity in printed e-textiles requires not only the right chemical pretreatment but also careful selection of textile architecture and printing orientation. The integration of these factors is essential for the scalable production of high-performance, reproducible, and industrially viable e-textiles using flexography.

Additional observations indicate that electrical resistance decreases with increasing grammage and yarn linear density (dtex), and with lower pretreatment pick-up values, while no consistent correlation was found for weft density or substrate thickness. Furthermore, the lowest resistance values occur when the printing direction, warp/weft orientation, and circuit pattern alignment coincide. Finally, no significant correlation was found between pretreatment absorption and conductivity, reinforcing that textile structure and surface characteristics are more influential factors for achieving low electrical resistance.

In summary, the physical structure of the textile substrate, particularly its regularity and compactness, is as critical as chemical pretreatment for achieving low electrical resistance and high-quality printed electronics. Lower surface roughness favors the formation of continuous conductive layers, whereas irregular textures generate micro-cracks that increase resistivity. Fabrics such as T4, characterized by structural uniformity, exhibit reduced variability and improved reproducibility, supporting industrial scalability and minimizing defect rates.

Flexography, when combined with optimized pretreatments and structurally favorable substrates, emerges as a promising technique for industrial-scale production of high-performance e-textiles.

## Figures and Tables

**Table 1:** Fabric characteristics (I): composition and ligament.

Fabric	Material	Ligament	Graphic Representation	Interlacing Coefficient (KL)
T1, T2, T3, T4	PES	Taffeta	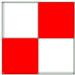	1
T5, T6, T7, T8	PES	Twill	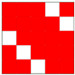	0.4

**Table 2:** Fabric characteristics (II): size and weight characteristics.

Fabric	Weft Density (Thread/cm)	Weft Yarn Count (dtex)	Thickness (µm)	Grammage (g/m^2^)
T1	10	333.3	515	154
T2	15	333.3	550	171
T3	10	666.7	622	191
T4	15	666.7	650	233
T5	10	333.3	705	160
T6	15	333.3	725	180
T7	10	666.7	744	222
T8	15	666.7	805	241

**Table 3:** PFI-RSA6004 Silver ink characteristics.

Ink Code	Density (g/mL)	Solids (%)	Viscosity (Pas)	Volume Resistivity (µΩ·cm)	Curing	Properties
PFI-RSA6004	2.25	60 (±2)	50–150 @1000 s^−1^	10–12	10–60 s 140 °C	- Fast Curing - Good Conductivity - Good Flexibility - Compatible with Polyester

**Table 4:** Printing Parameters.

Ink	Anilox Volume	Resolution	Printed Area	Speed	Curing
PFI-RSA6004—Silver Ink	11 cm^3^/m^2^	150 LPI	150 × 95 mm^2^	0.5 m/s	60 s, 140 °C

**Table 5:** Pick-up results for the different samples based on the used pretreatment.

	Hydrophobic Pretreatment (%)	Oleophobic Pretreatment (%)
T1	76.46	83.11
T2	72.87	77.02
T3	74.08	79.28
T4	64.73	66.98
T5	80.26	84.47
T6	78.80	87.51
T7	79.02	82.94
T8	74.39	78.92

**Table 6:** Drop situation results. A means highest repellence and D lowest repellence.

	Hydrophobic Pretreatment (%)	Oleophobic Pretreatment (%)
T1	A	A
T2	A	A
T3	A	A
T4	A	A
T5	A	A
T6	A	A
T7	A	A
T8	A	B

**Table 7:** Electrical Resistance (Ω) (mean ± standard deviation) relative to Printing Direction and Fabric Orientation (Warp and Weft).

	Untreated (UT)				Oleophobic Pretreatment (OT)				Hydrophobic Pretreatment (HT)			
	A-UT	B-UT	C-UT	D-UT	A-OT	B-OT	C-OT	D-OT	A-HT	B-HT	C-HT	D-HT
T1	--	--	--	--	--	--	--	--	--	204 ± 20	--	--
T2	13.7 ± 0.5	13.3 ± 0.4	14.3 ± 0.6	15.2 ± 0.7	3.15 ± 0.11	2.17 ± 0.10	4.50 ± 0.15	5.60 ± 0.18	13.1 ± 0.5	12.7 ± 0.4	13.5 ± 0.6	14.2 ± 0.7
T3	--	180 ± 10	--	--	--	17.8 ± 1.2	--	--	--	38,000 ± 28.3	--	--
T4	3.10 ± 0.15	2.24 ± 0.12	3.60 ± 0.18	3.80 ± 0.20	0.90 ± 0.05	0.52 ± 0.03	1.20 ± 0.06	1.80 ± 0.09	1.90 ± 0.08	1.80 ± 0.07	2.10 ± 0.10	2.40 ± 0.12
T5	146 ± 2	140 ± 2	147 ± 2	150 ± 2	260 ± 3.5	255 ± 3.5	265 ± 3.5	269 ± 3.5	11.2 ± 2	10.3 ± 2	11.9 ± 2	12.5 ± 2
T6	255 ± 2	254 ± 2	256 ± 2	257 ± 2	64.1 ± 1	63.5 ± 1	65.2 ± 1	66.7 ± 1	47.1 ± 1	46.6 ± 1	48.2 ± 1	49.1 ± 1
T7	12.5 ± 0.2	11.9 ± 0.2	12.8 ± 0.2	13.5 ± 0.2	11.2 ± 0.2	10.4 ± 0.2	12.1 ± 0.2	13.2 ± 0.2	46,300 ± 32.7	46,100 ± 32.5	47,100 ± 33.2	47,200 ± 33.3
T8	145,000 ± 1020	144,000 ± 1010	146,000 ± 1030	147,000 ± 1040	7.2 ± 0.2	6.76 ± 0.2	7.9 ± 0.2	8.9 ± 0.2	122,000 ± 860	121,000 ± 850	123,000 ± 870	124,000 ± 880

Note: Unspecified data is high impedance (open circuit).

**Table 8:** Color measurement. CIELAB.

Name	L*	a*	b*	ΔE*ab
Reference (untreated fabric)	92.3189	−0.7652	2.6213	
T2-UT	50.0492	0.1327	8.3465	42.665
T2-OT	52.7355	−0.607	9.9455	40.2556
T2-HT	54.7506	0.9541	9.2097	38.1804
T4-UT	52.5553	1.3045	9.4662	40.4015
T4-OT	53.3847	1.5468	9.0384	39.5271
T4-HT	54.7383	1.3473	10.0602	38.3679

## Data Availability

The original contributions presented in this study are included in the article. Further inquiries can be directed to the corresponding author.

## Abbreviations

The following abbreviations are used in this manuscript:

PCB	Printed Circuit Board
LPI	Lines Per Inch
UT	Untreated
HT	Hydrophobic Pretreatment
OT	Oleophobic Pretreatment
